# The ABILHAND‐23 Patient Reported Outcome Measure in Secondary Progressive Multiple Sclerosis: A Cross‐Sectional Analysis With the Nine Hole Peg Test

**DOI:** 10.1002/brb3.71101

**Published:** 2025-11-26

**Authors:** Sean Apap Mangion, Charles Wade, Tom Williams, Alessia Bianchi, Floriana De Angelis, Sarah Wright, Nevin John, Anisha Doshi, Alberto Calvi, Obioma Oraluzume, Maya Leibowitz, Guglielmo Vecchio, James Blackstone, Marie Braisher, Graziella Favarato, Jennifer Nicholas, Rachel Farrell, Jeremy Chataway, The UCL MS‐STAT2 Investigators

**Affiliations:** ^1^ Queen Square Multiple Sclerosis Centre, Department of Neuroinflammation, UCL Queen Square Institute of Neurology, Faculty of Brain Sciences University College London London UK; ^2^ Department of Medicine, School of Clinical Sciences Monash University Clayton Australia; ^3^ Department of Neurology Monash Health Clayton Australia; ^4^ Comprehensive Clinical Trials Unit University College London London UK; ^5^ Department of Statistics, Institute of Child Health University College London London UK; ^6^ London School of Hygiene and Tropical Medicine Centre of Global Change and Health Medical Statistics London UK; ^7^ Department of Neurorehabilitation The National Hospital for Neurology London UK; ^8^ National Institute for Health and Care Research, Biomedical Research Centre University College London Hospitals London UK

**Keywords:** clinical trials, outcome measures, PROMs, progressive

## Abstract

**Background:**

People with progressive multiple sclerosis (pwPMS), who typically have established lower limb dysfunction, experience greater disability from upper limb dysfunction (ULD). The 9‐hole peg test (9HPT) is the primary clinical measure for ULD but does not fully capture the patient experience. The ABILHAND‐23 is a well‐validated patient‐reported outcome measure (PROM) that evaluates bimanual ability in daily function. However, no large‐scale studies have assessed if the 9HPT reflects the individual ULD experience in pwPMS.

**Objectives:**

We sought to (van Munster et al. 2023) assess the associations between the ABILHAND‐23 and 9HPT, and (Huertas‐Hoyas et al. 2020) to assess the ability of the 9HPT and other relevant covariables to predict ABILHAND‐23 scores, using baseline data from the MS‐STAT2 trial, a phase 3 study on simvastatin for secondary progressive MS (SPMS).

**Methods:**

A cross‐sectional analysis of baseline data from the UCLH cohort of the MS‐STAT2 trial was performed using multiple linear regression to predict ABILHAND‐23 logit scores by 9HPT.

**Results:**

225 participants were analyzed. ABILHAND‐23 scores moderately correlated with the 9HPT (rho = 0.47). Regression analysis showed that better 9HPT performance modestly predicted ABILHAND‐23 logits (β = –0.05, SE 0.008, *p*‐value < 0.001).

**Conclusion:**

The 9HPT only modestly predicts the ABILHAND‐23 but does not fully capture the individual's daily disability experience, underscoring the value of patient‐reported outcome measures (PROMs) like the ABILHAND‐23 in clinical trials.

## Graphical abstract

This graphical abstract summarises the relationship between the 9‐Hole Peg Test (9HPT) and the ABILHAND‐23 in people with progressive multiple sclerosis (pwPMS). Baseline data from MS‐STAT2 (n = 225), showed that the 9HPT moderately correlated with ABILHAND‐23 scores (? = 0.47), but only modestly predicted patient‐reported functional ability. This highlights that timed assessments like the 9HPT do not fully capture the lived experience of upper limb disability, underscoring the complementary value of PROMs such as ABILHAND‐23 in trials and practice.

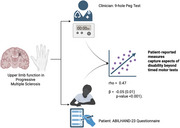

**Abbreviations**
9HPT: Nine‐hole peg test
EDSS: Expanded disability status scale
MS: Multiple sclerosis
PROM: Patient reported outcome measure
pwMS: People with multiple sclerosis
RRMS: Relapsing remitting multiple sclerosis
SD: Standard deviation
SE: Standard error
SPMS: Secondary progressive multiple sclerosis
T25FW: Timed 25‐foot walk
UCL: University College London
UCLH: University College London Hospital
ULD: Upper limb dysfunction

## 1 Introduction

ULD remains a major problem in MS, with contemporary series giving rates of up to 90% (van Munster et al. 2023, Huertas‐Hoyas et al. 2020, Solaro et al. 2023). Comprehensive assessment and measurement of it in clinical trials of pwPMS is crucial, as the traditional Expanded disability status scale (EDSS) is heavily influenced by walking ability in this cohort (Cadavid et al. 2017). The most established measure of ULD is the 9HPT (Feys et al. 2017). This measures the time taken to insert and remove nine pegs from a peg board; it is generally performed twice with each hand, and an average speed on both hands is used to assess upper limb function.

A 20% worsening in the average 9HPT speed is considered to be meaningful (van Winsen et al. 2010), reliably demonstrating worsening disability (Kragt et al. 2006), and shows less variation/measurement error in trials in PMS compared with the EDSS and timed 25‐foot walk (T25FW) (Koch et al. 2021). Practical limitations to its use include that it generally requires a participant to physically attend and for a suitably trained clinician to conduct the test.

Congruent with this, the importance of PROMs has evolved (Fiorini et al. 2015), from inexpensive and patient‐focused tools for monitoring clinical change to their potential utility as an outcome measure in clinical trials (Prada et al. 2021, Strijbis et al. 2022).

The ABILHAND questionnaire was originally developed as a semi‐structured interview to assess upper limb manual ability in people with rheumatoid arthritis using the Rasch analysis model (Penta et al. 1998), and has since been validated in numerous conditions, including MS (Cano et al. 2015). Differing versions with alternative numbers of items and response‐options exist including the ABILHAND‐23, having 23‐items and three potential answers (2 = “easy,” 1 = “difficult,” and 0 = “impossible”), and requiring 5–10 min to complete, was validated first in pwMS (Penta et al. 1998, Barrett et al. 2013), and ABILHAND‐56 which uses 56‐items, with 3 potential answers (“2 = easy,” “1 = difficult,” “0 = impossible”), and a version with 4 potential answers (“3 = easy,” “2 = difficult,” “1 = very difficult,” “0 = impossible”). The ordinal responses to each item are converted into a logit (log‐odds units) score of upper limb function, taking into account the relative difficulty of each question (item locations), which is held as being more psychometrically robust than the raw score (Hobart et al. 2007, Hobart and Cano 2009, Hobart et al. 2013, Barrett et al. 2015 Apr 1). These various forms of ABILHAND have been used in some drug and rehabilitation studies (see supplementary material) however, with inconsistent findings between clinical measurements of physical impairment and ABILHAND outcomes, highlighting the need for an assessment of the relationship in pwPMS.

Several smaller studies have assessed the correlation between ABILHAND and measures of ULD (Huertas‐Hoyas et al. 2020, Gatti et al. 2015, Savin et al. 2016, Boffa et al. 2020, Grange et al. 2021), with varying numbers of participants (the largest SPMS cohort being *n* = 59) (Grange et al. 2021). A recent meta‐analysis into the correlations between 9HPT and various ULD PROMS highlighted that they correlate better in studies with a mean or median EDSS of ≥ 6.0, and that ABILHAND‐23 shows a Pearson correlation with the dominant hand ranging between 0.23 to 0.46, and the non‐dominant hand between 0.04 and 0.12 (Grange et al. 2023). Its ability to predict 9HPT is unclear, though a small study of 30 individuals with either RRMS or SPMS (numbers not specified) demonstrated an ability to predict up to 46% of the variance in 9HPT dominant hand, and 33% of the non‐dominant hand (Martínez‐Piédrola et al. 2021), highlighting its potential use as a surrogate marker in clinical trials.

MS‐STAT2 is a multicenter, interventional, phase 3 randomized controlled trial assessing simvastatin versus placebo as a treatment in patients with SPMS, with the primary outcome being 6‐month confirmed disability worsening of EDSS (Blackstone et al. 2024). Eligible participants were 25–65 years of age with EDSS 4.0–6.5, with a confirmed diagnosis of SPMS and evidence of ongoing disability progression. The monitoring of ULD included the clinically collected 9HPT, as well as patient‐reported ABILHAND‐23 at the lead site.

This study cohort represents the largest SPMS group in which ABILHAND‐23 has been assessed in comparison to the well‐established and widely recognized 9HPT, with baseline data utilized from participants that took part in the MS‐STAT2 trial. Our aims were (van Munster et al. 2023) to assess the relationship between 9HPT and ABILHAND‐23, and (Huertas‐Hoyas et al. 2020) to assess the ability of the 9HPT to predict the subjective individual experience of ULD in the form of ABILHAND‐23 raw total scores and Rasch analysis logit scores.

## 2 Patients and Methods

### 2.1 Patients

Participants included in this analysis were recruited into the MS‐STAT2 randomized controlled trial (NCT03387670) at the trial lead site, University College London Hospital (UCLH). All participants gave written informed consent. The MS‐STAT2 trial was approved by the NHS national research ethics committee (London Westminster Research Ethics Committee, 09/10/2017, ref. 17/LO/1509) and conducted according to the Declaration of Helsinki (World Medical Association).

From a total of 315 participants recruited at the UCL MS‐STAT2 site, 225 consented to take part in the ABILHAND‐23 study and were included in the analysis. Inclusion criteria for the MS‐STAT2 study required a diagnosis of SPMS with evidence of disability progression and an EDSS between 4.0 to 6.5 (inclusive) (Blackstone et al. 2024).

### 2.2 Baseline Assessments

The EDSS and 9HPT were performed by trained neurologists. The 9HPT is performed by measuring the time taken to insert nine pegs into slots and extract them again twice with the non‐dominant, then twice with the non‐dominant hand, with either being averaged, and the final score being the average of these two measures. A normal performance is seen to be <1 9 s for the dominant hand and < 21 s for the non‐dominant hand (Feys et al. 2017, Koch et al. 2023). The 23‐item ABILHAND was administered in person or via telephone, with responses to each item being either: impossible (0), difficult (van Munster et al. 2023), or easy (Huertas‐Hoyas et al. 2020). The results were summed to generate a total score (maximal 46‐points, reflecting unimpaired upper limb function), and individual responses were converted into a linear measure of manual ability using Rasch analysis via an online RUMM2030 tool, which is able to account for missing responses (http://www. rehab‐scales.org/abilhand‐rasch‐analysis‐chronic‐stroke.html) (Andrich et al. 2010, Kahraman 2018).

### 2.3 Statistical Analysis

9HPT outcomes demonstrated a significant positive skew, confirmed on Shapiro‐Wilks and Anderson‐Darling testing. ABILHAND‐23 raw scores and logit scores demonstrated a significant negative skew, most notable on Anderson‐Darling testing; normalcy could not be achieved by transformative measures.

The analysis included (van Munster et al. 2023) assessing the relationship between ABILHAND‐23 and 9HPT, which was conducted using Spearman correlation due to non‐normalcy, and (Huertas‐Hoyas et al. 2020) assessing the ability of 9HPT to account for ABILHAND‐23 scores in a multiple linear regression model. The combined mean of either hand function from the 9HPT was utilised rather than either hand, as is standard in PMS clinical trials (Feys et al. 2017, Koch et al. 2023), and because the ABILHAND questionnaire assesses capacity on bimanual tasks. The following covariates were included because of their potential to influence ABILHAND‐23 outcomes in the form of disability progression over time, experience of disabilities over time, and self‐perception, as highlighted in previous studies (Martínez‐Piédrola et al. 2021): age at randomization, duration of progressive illness, and the presence of self‐reported depression. A further model including an interaction term for EDSS severity (score ≤ 5.5 vs. ≥ 6.0) was included due to its potential impact on both ABILHAND‐23 and 9HPT scores. Handedness was not included as the ABILHAND‐23 assesses bimanual ability.

Assumptions of linearity, residual homoscedasticity, and multicollinearity were checked and shown in the supplementary material.

## 3 Results

Baseline measurements for the 225 participants are detailed in Table 1. 170 participants were female (75.6%), with a mean age of 54.5 years (SD: 7.32, range 32 to 65 years), mean MS onset of 24.3 years (SD: 9.2, range 4.5 to 47.4 years), mean MS progression duration of 7.8 years (SD 4.6, range 2.0 to 23.81 years), and a median EDSS of 6.0 (range 4.0 to 6.5). The mean total ABILHAND‐23 score was 32.6 (SD 9.5, range 8 to 46), mean ABILHAND‐23 logit were 1.89 (SD 1.94, range ‐2.17 to 6.017), mean dominant hand 9HPT was 34.08s (SD 23.88, range 17.1 to 221.5s), and mean non‐dominant hand 9HPT was 34.52s (SD 19.40, range 16.8 to 224.0 s).

**TABLE 1:** Demographics and outcomes (*n* = 225).

Gender Female Male	170 (75.6%) 55 (24.4%)
Age (years) Mean SD (range)	54.5 7.32 (32.0 to 65.0)
MS onset duration (years) Mean SD (range)	24.3 9.2 (4.5 to 47.4)
MS progression duration (years) Mean SD (range)	7.8 4.6 (2.0 to 23.81)
EDSS Median Range	6.0 4.0 to 6.5
9HPT (s) Dominant hand Mean SD (range) Non‐dominant hand Mean SD (range)	34.08 23.88 (17.1 to 221.5) 34.52 19.40 (16.8 to 224.0)
ABILHAND‐23 total score* Median IQR (range)	37 117.25 (8 to 46)
ABILHAND‐23 logit Mean IQR (range)	1.91 2.63 (‐2.179 to 6.017)

*Note*: *To limit bias from unanswered/missing items in the ABILHAND‐23 total score, these values only reflect results from participants who answered all the questionnaire items (*n* = 156). This does not apply to ABILHAND‐23 logit as it accounts for missing responses.

**FIGURE 1:**
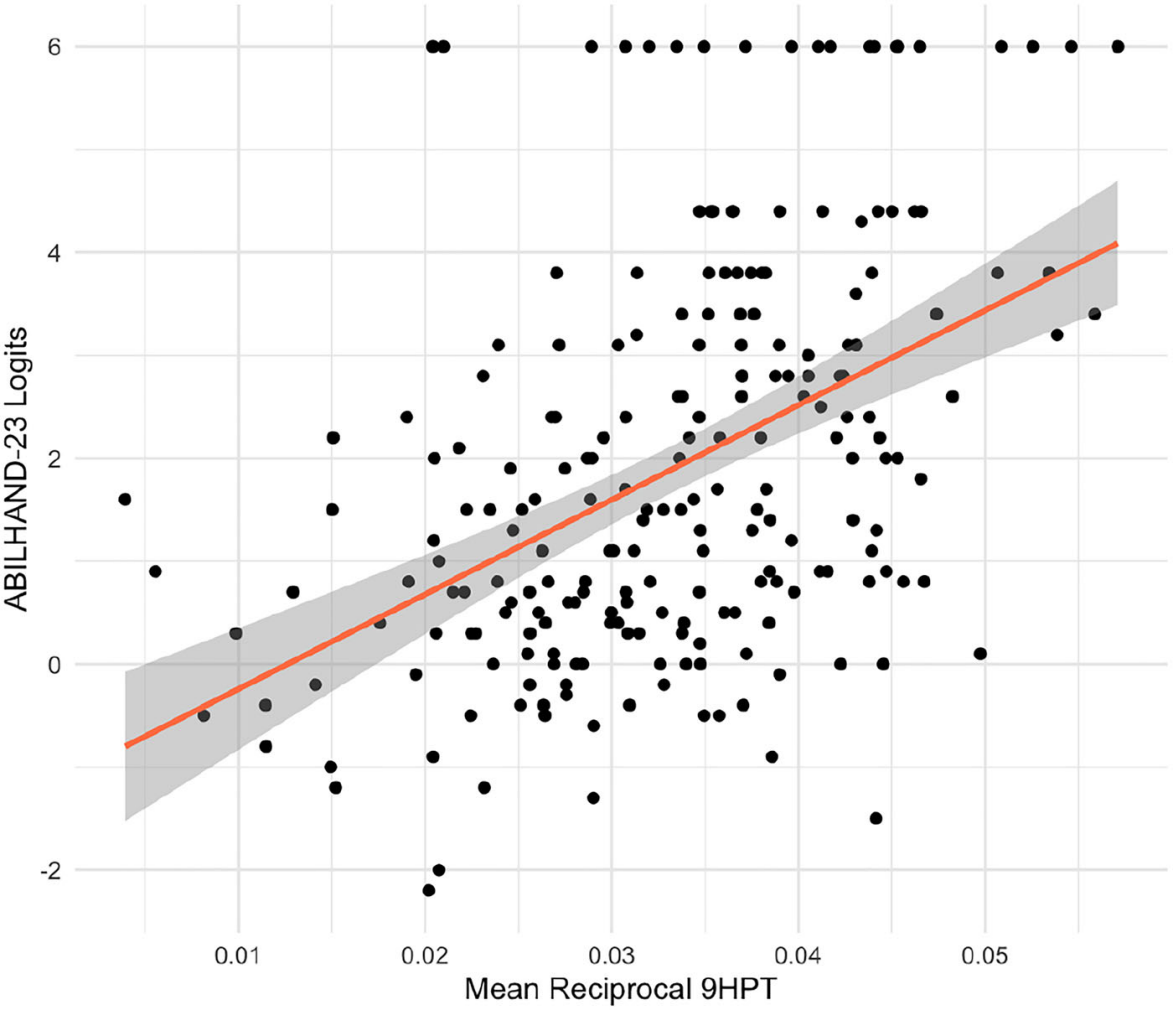
Scatterplots of ABLHAND‐23 total scores and logit with reciprocal 9HPT. The solid red line represents the fitted linear regression line, and the grey‐shaded area the 95% confidence interval.

ABILHAND‐23 total scores and logits correlated moderately and similarly with the mean reciprocal 9HPT (respectively: Spearman's rho 0.56, *p*‐value < 0.001, and rho 0.47, *p*‐value < 0.001), demonstrated in Figure 1.

The multiple linear regression analysis model found that bimanual mean 9HPT, age, SPMS disease duration, and depression together explained 17% of the variance of ABILHAND‐23 logit scores (adjusted *R^2^* = 0. 0.1665, *p*‐value < 0.001). Improved combined mean 9HPT was significantly associated with ABILHAND‐23 logit score, with every 20 s worse performance of combined mean 9HPT predicting a 1‐point decrease in the ABILHAND‐23 logit score (β = –0.05, SE 0.008, *p*‐value < 0.001). The presence depression predicted a worse ABILHAND logit score (β = –0.78, SE 0.27, *p*‐value = 0.004). There was no significant difference between the EDSS severity groups (low < = 5.5 versus high > = 6.0) in the relationship between 9HPT and ABILHAND‐23 logit scores (*p* = 0.68 interaction test, see supplementary materials for details).

## 4 Discussion

We demonstrate, with the largest reported SPMS cohort assessing ABILHAND‐23 and 9HPT, that a significant amount of the day‐to‐day ULD experienced by pwPMS is not accounted for by the 9HPT, nor indeed by other often utilized measures in clinical trials (duration of illness, age, and baseline EDSS) related to final outcomes of interest, which may reflect additional elements such as fatigue or psychosocial factors that can impact PROs (Young et al. 2024). This supports the use of PROMS as a complimentary outcome measure in clinical trials to traditional clinical assessments, given that they reflect outcomes that matter most to patients (Brichetto, 2020) and are of value for healthcare stakeholders (Zaratin et al. 2022).

We take this view in the context of the MS walking scale 12 (MSWS‐12) questionnaire (Hobart et al. 2003), which was similarly developed using robust techniques and has demonstrated an ability to detect significant change in pwPMS (Baert et al. 2018), allowing its utility in clinical trials as a joint primary outcome measure (Hobart et al. 2019) or important secondary outcome measures (supplementary material). However, as with other PROMs (Ontaneda et al. 2024), consensus on the collection of ABILHAND, including which version, and what would constitute significant change over time at both an individual and trial level, for the different MS subtypes needs to be established.

With this in mind, a longitudinal analysis of ABILHAND‐23 will be undertaken to explore its potential utility in remote trials of PMS or as an interim measure in PMS multi‐arm multi‐stage trials, such as the OCTOPUS trial (ISRCTN140448364).

The strengths of this study are that it is a large cohort of well‐described pwSPMS in the context of a phase 3 clinical trial, conducted prior to any treatment initiation and with blinding to the final trial result. Limitations include that it does not include individuals with higher EDSS (> 6.5) and its cross‐sectional nature.

## Author Contributions

**Sean Apap Mangion**: conceptualization, methodology, software, formal analysis, investigation, visualization, writing – original draft, writing – review and editing, validation, data curation. **Charles Wade**: writing – review and editing, data curation. **Tom Williams**: data curation, writing – review and editing. **Alessia Bianchi**: writing – review and editing, data curation. **Floriana De Angelis**: writing – review and editing, data curation. **Sarah Wright**: data curation, writing – review and editing. **Nevin John**: writing – review and editing, data curation. **Anisha Doshi**: writing – review and editing, data curation. **Alberto Calvi**: data curation, writing – review and editing. **Obioma Oraluzume**: data curation, writing – review and editing. **Maya Leibowitz**: data curation, writing – review and editing. **Guglielmo Vecchio**: writing – review and editing, data curation. **James Blackstone**: funding acquisition, writing – review and editing, resources, project administration, investigation. **Marie Braisher**: funding acquisition, writing – review and editing, project administration, resources. **Graziella Favarato**: formal analysis, writing – review and editing, conceptualization. **Jennifer Nicholas**: conceptualization, writing – review and editing, validation, formal analysis. **Rachel Farrell**: writing – review and editing, conceptualization, methodology. **Jeremy Chataway**: supervision, conceptualization, funding acquisition, writing – review and editing, project administration, resources, validation, methodology.

## Funding

This trial has been cofunded by the National Institute for Health Research (NIHR) Health Technology Assessment (HTA) Programme—HTA Project: 15/57/143, the UK Multiple Sclerosis Society and the National Multiple Sclerosis Society in the USA.

## Ethics Statement

The MS‐STAT2 trial was approved by the NHS national research ethics committee (London Westminster Research Ethics Committee, 09/10/2017, ref: 17/LO/1509).

## Consent

All participants gave written consent.

## Conflicts of Interest

In the last 3 years, JC has received support from the Health Technology Assessment (HTA) Programme (National Institute for Health Research, NIHR), the UK MS Society, the US National MS Society and the Rosetrees Trust. He is supported in part by the NIHR University College London Hospitals (UCLH) Biomedical Research Centre, London, UK. He has been a local principal investigator for a trial in MS funded by MS Canada. A local principal investigator for commercial trials funded by: Ionis and Roche; and has taken part in advisory boards/consultancy for Biogen, Contineum Therapeutics, InnoCare, Lucid, Merck, NervGen, Novartis and Roche. NJ is a principal investigator on commercial MS trials sponsored by Roche, Novartis and Biogen. He has received speakers honoraria from Merck and travel congress sponsorship from Novartis.

## Supporting information




**Supplementary Material**: brb371101‐sup‐0001‐SuppMat.docx

**Supplementary Table**: brb371101‐sup‐0002‐Table.docx

## Data Availability

The data that support the findings of this study are available on request from the corresponding author. The data are not publicly available due to privacy or ethical restrictions.

## References

[brb371101-bib-0001] Andrich, D. , B. Sheridan , and G. Luo . 2010. RUMM2030: A Windows Program for the Analysis of Data According to Rasch Unidimensional Models for Measurement. Perth, WA: RUMM Laboratory Pty Ltd;.

[brb371101-bib-0002] Baert, I. , T. Smedal , A. Kalron , et al. 2018. “Responsiveness and Meaningful Improvement of Mobility Measures Following MS Rehabilitation.” Neurology 91, no. 20: 1880–1892. 10.1212/WNL.0000000000006532 30333161

[brb371101-bib-0003] Barrett, L. , S. Cano , J. Zajicek , and J. Hobart . 2015. “Lending a Hand: Can DASH Items Help ABILHAND Improve Manual Ability Measurement in Multiple Sclerosis?” Multiple Sclerosis 21, no. 5: 612–621. 25583836 10.1177/1352458514549396

[brb371101-bib-0004] Barrett, L. E. , S. J. Cano , J. P. Zajicek , and J. C. Hobart . 2013. “Can the ABILHAND Handle Manual Ability in MS?” Multiple Sclerosis Journal 19, no. 6: 806–815. 23095289 10.1177/1352458512462919

[brb371101-bib-0005] Blackstone, J. , T. Williams , J. M. Nicholas , et al. 2024. “Evaluating the Effectiveness of simvastatin in Slowing the Progression of Disability in Secondary Progressive Multiple Sclerosis (MS‐STAT2): Protocol for a Multicentre, Randomised Controlled, Double‐Blind, Phase 3 Clinical Trial in the UK.” BMJ Open 14, no. 9:e086414. 10.1136/bmjopen-2024-086414 PMC11409264 39284697

[brb371101-bib-0006] Boffa, G. , A. Tacchino , E. Sbragia , et al. 2020. “Preserved Brain Functional Plasticity After Upper Limb Task‐Oriented Rehabilitation in Progressive Multiple Sclerosis.” European Journal of Neurology 27, no. 1: 77–84. 31419353 10.1111/ene.14059

[brb371101-bib-0007] Brichetto, G. 2020. “We Should Monitor Our Patients With Wearable Technology Instead of Neurological Examination—Commentary.” Multiple Sclerosis Journal 26, no. 9: 1028–1030. 32669039 10.1177/1352458520930985

[brb371101-bib-0008] Cadavid, D. , J. A. Cohen , and M. S. Freedman . 2017. “The EDSS‐Plus, an Improved Endpoint for Disability Progression in Secondary Progressive Multiple Sclerosis.” Multiple Sclerosis 23, no. 1: 94–105. 27003945 10.1177/1352458516638941

[brb371101-bib-0009] Cano, S. , S. Cleanthous , P. Marquis , et al. 2015. “Measuring Upper Limb Function in Multiple Sclerosis: Enhancing the Abilhand's Performance.” Value in Health 18, no. 3: A24.

[brb371101-bib-0010] Feys, P. , I. Lamers , G. Francis , et al. 2017. “The Nine‐Hole Peg Test as a Manual Dexterity Performance Measure for Multiple Sclerosis.” Multiple Sclerosis Journal 23, no. 5: 711–720. 28206826 10.1177/1352458517690824 PMC5405844

[brb371101-bib-0011] Fiorini, S. , A. Verri , A. Tacchino , M. Ponzio , G. Brichetto , and A. Barla . 2015. “A Machine Learning Pipeline for Multiple Sclerosis Course Detection From Clinical Scales and Patient Reported Outcomes.” in 2015 37th Annual International Conference of the IEEE Engineering in Medicine and Biology Society (EMBC) . 10.1109/EMBC.2015.7319381 26737281

[brb371101-bib-0012] Gatti, R. , A. Tettamanti , S. Lambiase , P. Rossi , and M. Comola . 2015. “Improving Hand Functional Use in Subjects With Multiple Sclerosis Using a Musical Keyboard: A Randomized Controlled Trial.” Physiotherapy Research International 20, no. 2: 100–107. 25045035 10.1002/pri.1600

[brb371101-bib-0013] Grange, E. , D. Marengo , R. Di Giovanni , et al. 2021. “Italian Translation and Psychometric Validation of the ABILHAND‐26 and Its Correlation With Upper Limb Objective and Subjective Measures in Multiple Sclerosis Subjects.” Multiple Sclerosis and Related Disorders 55: 103160. 34320388 10.1016/j.msard.2021.103160

[brb371101-bib-0014] Grange, E. , C. Solaro , R. Di Giovanni , and D. Marengo . 2023. “The Correlation Between 9‐HPT and Patient‐Reported Measures of Upper Limb Function in Multiple Sclerosis: A Systematic Review and Meta‐Analysis.” Journal of Neurology 9: 4179–4191. 10.1007/s00415-023-11801-3 PMC10421783 37294322

[brb371101-bib-0015] Hobart, J. , and S. Cano . 2009. “Improving the Evaluation of Therapeutic Interventions in Multiple Sclerosis: the Role of New Psychometric Methods.” Health Technology Assessment 13, no. 12: 1–177. 10.3310/hta13120 19216837

[brb371101-bib-0016] Hobart, J. , S. Cano , H. Posner , et al. 2013. “Putting the Alzheimer's Cognitive Test to the Test II: Rasch Measurement Theory.” Alzheimer's and Dementia 9, no. 1: S10–S20. 10.1016/j.jalz.2012.08.006 23253779

[brb371101-bib-0017] Hobart, J. , T. Ziemssen , P. Feys , et al. 2019. “Assessment of Clinically Meaningful Improvements in Self‐Reported Walking Ability in Participants With Multiple Sclerosis: Results From the Randomized, Double‐Blind, Phase III ENHANCE Trial of Prolonged‐Release Fampridine.” CNS Drugs 33, no. 1: 61–79. https://link.springer.com/10.1007/s40263‐018‐0586‐5. 30535670 10.1007/s40263-018-0586-5 PMC6328522

[brb371101-bib-0018] Hobart, J. C. , S. J. Cano , J. P. Zajicek , and A. J. Thompson . 2007. “Rating Scales as Outcome Measures for Clinical Trials in Neurology: Problems, Solutions, and Recommendations.” Lancet Neurology 6, no. 12: 1094–1105. 18031706 10.1016/S1474-4422(07)70290-9

[brb371101-bib-0019] Hobart, J. C. , A. Riazi , D. L. Lamping , et al. 2003. “Measuring the Impact of MS on Walking Ability: The 12‐Item MS Walking Cale (MSWS‐12).” Neurology 60, no. 1: 31–36. 12525714 10.1212/wnl.60.1.31

[brb371101-bib-0020] Huertas‐Hoyas, E. , N. Máximo‐Bocanegra , C. Diaz‐Toro , et al. 2020. “A Descriptive Cross‐Sectional Study of Manipulative Dexterity on Different Subtypes of Multiple Sclerosis.” Occupational Therapy International 2020: 6193938. 32425718 10.1155/2020/6193938 PMC7211248

[brb371101-bib-0021] Kahraman, T. 2018. “Performance Measures for Upper Extremity Functions in Persons With Multiple Sclerosis.” Noropsikiyatri Arsivi 55: S41–S45. 30692854 10.29399/npa.23317 PMC6278617

[brb371101-bib-0022] Koch, M. W. , J. Mostert , P. Repovic , J. D. Bowen , B. Uitdehaag , and G. Cutter . 2021. “Reliability of Outcome Measures in Clinical Trials in Secondary Progressive Multiple Sclerosis.” Neurology 96, no. 1: e111–e120. 33106389 10.1212/WNL.0000000000011123

[brb371101-bib-0023] Koch, M. W. , P. Repovic , J. Mostert , et al. 2023. “The Nine Hole Peg Test as an Outcome Measure in Progressive MS Trials.” Multiple Sclerosis and Related Disorders 69: 104433. 36462470 10.1016/j.msard.2022.104433

[brb371101-bib-0024] Kragt, J. J. , F. A. van der Linden , J. M. Nielsen , B. M. Uitdehaag , and C. H. Polman . 2006. “Clinical Impact of 20% Worsening on Timed 25‐Foot Walk and 9‐Hole Peg Test in Multiple Sclerosis.” Multiple Sclerosis Journal 12, no. 5: 594–598. 17086905 10.1177/1352458506070768

[brb371101-bib-0025] Martínez‐Piédrola, R. M. , C. García‐Bravo , E. Huertas‐Hoyas , et al. 2021. “The Influence of Self‐Perception on Manipulative Dexterity in Adults With Multiple Sclerosis.” Occupational Therapy International 2021: 5583063. 34483781 10.1155/2021/5583063 PMC8384504

[brb371101-bib-0026] Ontaneda, D. , R. J. Fox , and J. Chataway . 2024. “Clinical Trials in Progressive Multiple Sclerosis: Lessons Learned and Future Perspectives.” Lancet Neurology 14, no. 2: 208–223. 10.1016/S1474-4422(14)70264-9 PMC4361791 25772899

[brb371101-bib-0027] Penta, M. , J. L. Thonnard , and L. Tesio . 1998. “ABILHAND: A Rasch‐Built Measure of Manual Ability.” Archives of Physical Medicine and Rehabilitation 79, no. 9: 1038–1042. 9749680 10.1016/s0003-9993(98)90167-8

[brb371101-bib-0028] Prada, V. , A. Tacchino , J. Podda , et al. 2021. “MaM‐36 and ABILHAND as Outcome Measures of multiple Sclerosis Hand Disability: an Observational Study.” European Journal of Physical and Rehabilitation Medicine 57, no. 4: 520–526. 33305546 10.23736/S1973-9087.20.06446-1

[brb371101-bib-0029] Savin, Z. , I. Lejbkowicz , L. Glass‐Marmor , I. Lavi , S. Rosenblum , and A. Miller . 2016. “Effect of Fampridine‐PR (Prolonged Released 4‐aminopyridine) on the Manual Functions of Patients With Multiple Sclerosis.” Journal of the Neurological Sciences 360: 102–109. 26723984 10.1016/j.jns.2015.11.035

[brb371101-bib-0030] Solaro, C. , R. Di Giovanni , E. Grange , et al. 2023. “Correlation Between Patient‐Reported Manual Ability and Three Objective Measures of Upper Limb Function in People With Multiple Sclerosis.” European Journal of Neurology 30, no. 1: 172–178. 36086993 10.1111/ene.15560 PMC10087787

[brb371101-bib-0031] Strijbis, E. M. M. , P. Repovic , J. Mostert , et al. 2022. “The MSIS‐29 and SF‐36 as Outcomes in Secondary Progressive MS Trials.” Multiple Sclerosis Journal 28, no. 10: 1606–1619. 35876467 10.1177/13524585221105465 PMC9315187

[brb371101-bib-0032] van Munster, C. E. P. , J. Burggraaff , S. Steinheimer , et al. 2023. “Assessment of Multiple Aspects of Upper Extremity Function Independent From Ambulation in Patients With Multiple Sclerosis.” International Journal of MS Care 25, no. 5: 226–232. 37720262 10.7224/1537-2073.2021-069 PMC10503816

[brb371101-bib-0033] van Winsen, L. M. , J. J. Kragt , E. L. Hoogervorst , C. H. Polman , and B. M. Uitdehaag . 2010. “Outcome Measurement in Multiple Sclerosis: Detection of Clinically Relevant Improvement.” Multiple Sclerosis Journal 16, no. 5: 604–610. 20086019 10.1177/1352458509359922

[brb371101-bib-0034] World Medical Association . Declaration of Helsinki: Ethical Principles for Medical Research Involving Human Subjects. https://www.wma.net/policies‐post/wma‐declaration‐of‐helsinki‐ethical‐principles‐for‐medical‐research‐involving‐human‐subjects/.

[brb371101-bib-0035] Young, C. A. , D. J. Rog , B. Sharrack , et al. 2024. “Physical and Psychological Aspects of Multiple Sclerosis: Revisiting the Multiple Sclerosis Impact Scale (MSIS‐29).” Multiple Sclerosis Journal 30, no. 13: 1630–1641. 39474866 10.1177/13524585241288393 PMC11568641

[brb371101-bib-0036] Zaratin, P. , P. Vermersch , M. P. Amato , et al. 2022. “The Agenda of the global patient Reported Outcomes for Multiple Sclerosis (PROMS) Initiative: Progresses and Open Questions.” Mult Sclerosis and Related Disorders 61: 103757. 10.1016/j.msard.2022.103757 35367873

