# Correction to “The ABILHAND‐23 Patient Reported Outcome Measure in Secondary Progressive Multiple Sclerosis: A Cross‐Sectional Analysis with the Nine Hole Peg Test”

**DOI:** 10.1002/brb3.71920

**Published:** 2026-11-10

Apap Mangion, S., C. Wade,  T. Williams, et al.  2025. “The ABILHAND‐23 Patient Reported Outcome Measure in Secondary Progressive Multiple Sclerosis: A Cross‐Sectional Analysis With the Nine Hole Peg Test.” *Brain and Behavior* 15, no. 12: e71101. https://doi.org/10.1002/brb3.71101

The name of the tenth author was misspelled and has been updated from Obioma Oraluzume to Obioma Orazulume.

The online version of this article has been corrected accordingly.

We apologize for this error.

