# Supplementary material for: The ABILHAND‐23 Patient Reported Outcome Measure in Secondary Progressive Multiple Sclerosis: A Cross‐Sectional Analysis With the Nine Hole Peg Test
Source: Brain Behav. 2025 Nov 26;15(12):e71101. doi: 10.1002/brb3.71101 (PMC12657254; doi:10.1002/brb3.71101)
Supplement: Supplementary file 1 — Supplementary Material: brb371101‐sup‐0001‐SuppMat.docx [file BRB3-15-e71101-s002.docx]

***Supplementary Material***

1. MS-STAT2 Investigators
2. ABILHAND-23 and Nine Hole Peg Test Distributions
3. Multiple Linear Regression Models
4. EDSS Group Multiple Linear Regression Model
5. **MS-STAT2 Investigators**

Jeremy Chataway, Thomas Williams, Nevin John, Floriana De Angelis, Alberto Calvi, Alessia Bianchi, Sarah Wright, Madiha Shatila, Anisha Doshi, Wallace Brownlee, Claudia A M Gandini Wheeler-Kingshott, Frederik Barkhof, Olga Ciccarelli, Jonathan Stutters, Ferran Prados Carrasco, Antonio Ricciardi, Marios Yiannakas, David MacManus, Megan Wynne, Marie Braisher (Queen Square Multiple Sclerosis Centre, University College London and University College London Hospitals NHS Foundation Trust, London, UK);

James Blackstone, Leanne Hockey, Josephine Parker, Jennifer Flight (Comprehensive Clinical Trials Unit [CCTU], Institute of Clinical Trials and Methodology, University College London, London, UK);

Chris Frost, Jennifer Nicholas (Centre for Statistical Methodology, London School of Hygiene and Tropical Medicine, London, UK);

Stuart Nixon and Judy Beveridge (patient representatives);

Siddharthan Chandran, Peter Connick, Dawn Lyle (Anne Rowling Regenerative Neurology Clinic, The University of Edinburgh, Royal Infirmary of Edinburgh, NHS Lothian, Edinburgh, UK);

Ian Galea, Elisabeth Jarman (University Hospital Southampton NHS Foundation Trust, Southampton, UK);

Helen Ford, Linford Fernandes, Maruthi Vinjam (Leeds Teaching Hospitals NHS Trust, Leeds, UK);

Sue Pavitt (Dental Translational and Clinical Research Unit, University of Leeds, Leeds, UK);

Basil Sharrack, David Paling (Sheffield Teaching Hospitals NHS Foundation Trust, Sheffield, UK);

Abdullah Shehu, Tarunya Arun, Mohamed Belhag (University Hospitals Coventry & Warwickshire NHS Trust, Coventry, UK);

Owen Pearson, Gillian Ingram, Christopher Rickards (Swansea Bay University Health Board, Swansea, UK);

Gavin McDonnell, Stella Hughes (Belfast Health and Social Care Trust, Belfast, UK);

Cord Spilker (Bradford Teaching Hospitals Foundation Trust, Bradford, UK);

Leonora Fisniku, Julia Aram (Brighton and Sussex University Hospitals NHS Trust, Brighton, UK);

Claire Rice (North Bristol NHS Trust, Bristol, UK);

Stefano Pluchino, Luca Peruzzotti-Jametti (Cambridge University Hospitals NHS Foundation Trust, Cambridge, UK)

Sreedharan Harikrishnan, Nikki Guck (East Kent Hospitals University NHS Foundation Trust, Canterbury, UK);

Neil Robertson, Emma Tallantyre (University Hospital of Wales, Cardiff, UK);

Timothy Harrower (Royal Devon University Healthcare NHS Foundation Trust, Exeter, UK);

Paul Gallagher (NHS Greater Glasgow and Clyde, Glasgow, UK);

Fayyaz Ahmed (Hull University Teaching Hospitals NHS Trust, Hull, UK);

Carolyn Young, Heike Arndt (The Walton Centre NHS Foundation Trust, Liverpool, UK);

Eli Silber (Lewisham and Greenwich NHS Trust, London, UK);

Richard Nicholas (Imperial College Healthcare NHS Trust, London, UK);

Martin Duddy (Royal Victoria Infirmary, The Newcastle upon Tyne Hospital NHS Foundation Trust, Newcastle, UK);

Martin Lee (Norfolk and Norwich University Hospitals NHS Foundation Trust, Norwich, UK);

Nikos Evangelou, Christopher Allen (Nottingham University Hospital NHS Trust, Nottingham, UK);

Matthew Craner, Ruth Geraldes (Oxford University Hospitals NHS Foundation Trust, Oxford, UK);

Jeremy Hobart (University Hospitals Plymouth NHS Trust, Plymouth, UK);

Charles Hillier (University Hospitals Dorset NHS Foundation Trust, Poole, UK);

Suresh Chhetri (Lancashire Teaching Hospitals NHS Foundation Trust, Preston, UK);

Miriam Mattoscio, Abhijit Chaudhuri (Barking, Havering and Redbridge University Hospitals NHS Trust, Romford, UK);

Seema Kalra (University Hospitals of North Midlands NHS Trust, Stoke-on-Trent, UK);

Agne Straukiene (Torbay & South Devon NHS Foundation Trust, Torbay, UK).

1. **ABILHAND-23 and Nine Hole Peg Test Distributions**

**
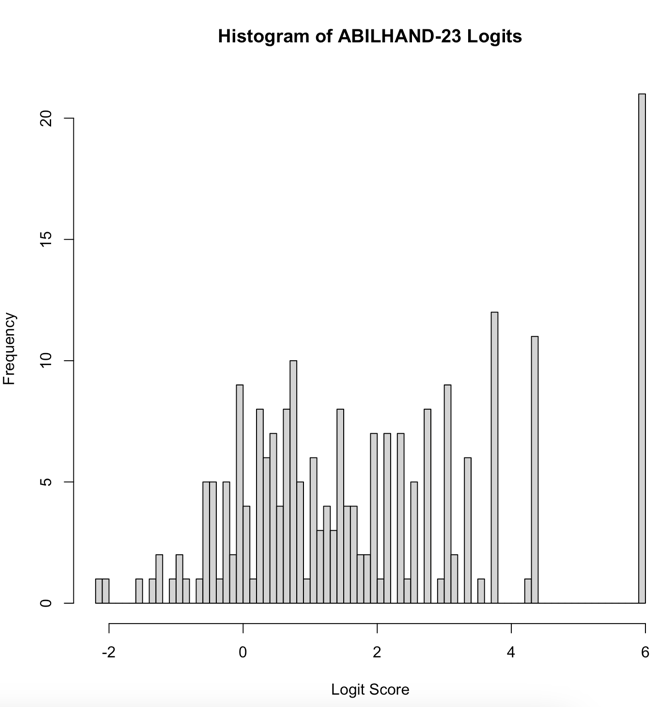

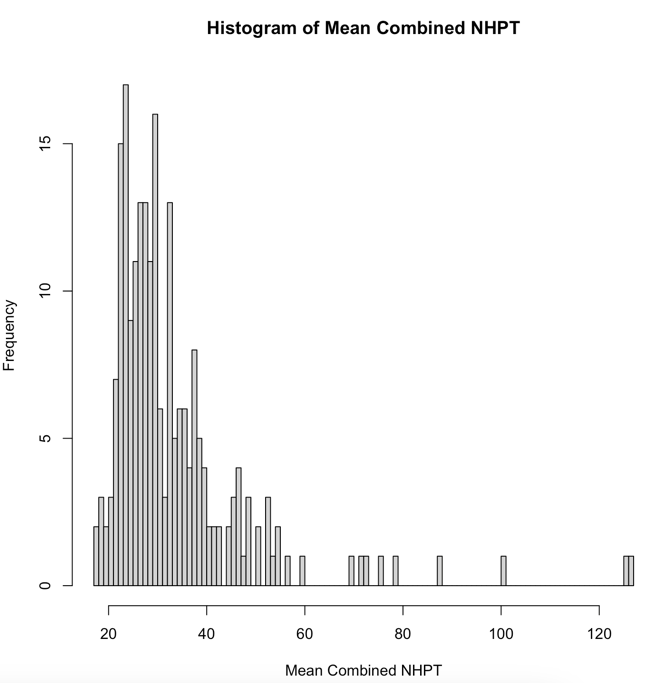
**

1. **Simple Multiple Linear Regression Model**

| Predictor | Estimate | Std. Error | t-value | p-value |
| --- | --- | --- | --- | --- |
| (Intercept) | 3.873009 | 0.308876 | 12.539 | < 2e-16 *** |
|  |  |  |  |  |
| Mean Combined NHPT | -0.049788 | 0.008174 | -6.091 | 5.21e-09 *** |
| AGE (centred) | -0.005211 | 0.017745 | -0.294 | 0.76929 |
| progressive Disease Duration (centred) | 0.024027 | 0.028711 | 0.837 | 0.40362 |
| depression | -0.780102 | 0.271574 | -2.873 | 0.00449 ** |

**Significance codes: *** p < 0.001, ** p < 0.01, * p < 0.05

#### Model Performance

- **Residual Standard Error**: 1.765 on 212 degrees of freedom
- **Multiple R-squared**: 0.182
- **Adjusted R-squared**: 0.1665
- **F-statistic**: 11.79 on 4 and 212 DF
- **p-value**: 1.149e-08
- **AIC**: 869.4138
- **BIC**: 889.6931

Model Fit and Assumptions

- **Residuals**: The distribution of the residuals was examined through plots and histograms. The residuals appeared to be approximately normally distributed, with the following summary statistics:
  - **Minimum**: -4.1173
  - **1st Quartile**: -1.2725
  - **Median**: -0.2278
  - **3rd Quartile**: 1.0183
  - **Maxmum**: 4.6708


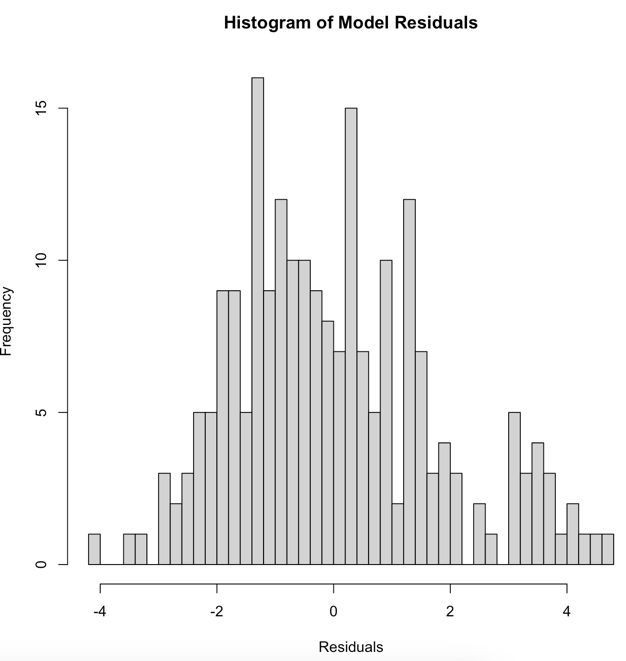

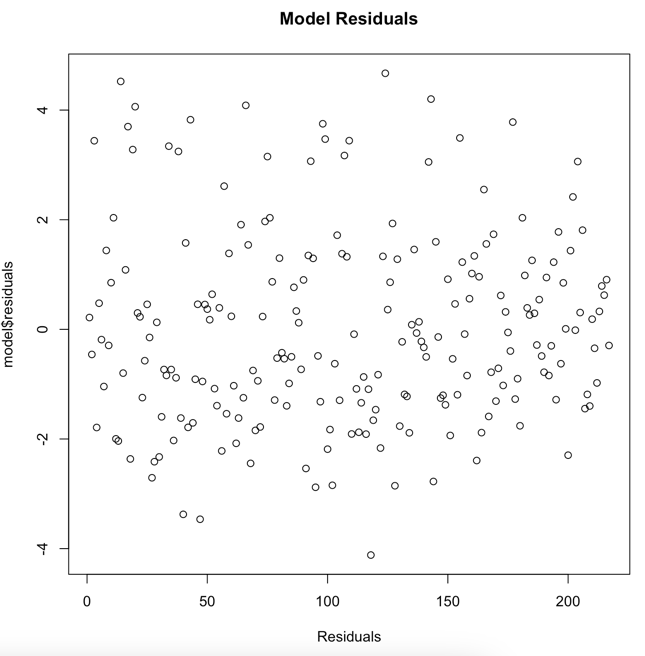


- **Variance Inflation Factor (VIF)**: Multicollinearity was assessed using VIF values, all of which were below 5, indicating no significant multicollinearity among the predictors:
  - Mean Combined NHPT: 1.058637
  - Age (centred): 1.101829
  - Progressive Disease Duration (centred): 1.088475
  - Depression: 1.016685

1. **Multilinear Regression Model with Interaction term**

| Predictor | Estimate | Std. Error | t-value | p-value |
| --- | --- | --- | --- | --- |
| (Intercept) | 4.179561 | 0. 516713 | 8.089 | 4.77e-14 *** |
|  |  |  |  |  |
| Mean Combined NHPT | -0.053124 | 0.015421 | -3.445 | 0.00069 *** |
| EDSS (≥6.0) | -0.598645 | 0.626425 | -0.956 | 0.34035 |
| AGE (centred) | -0.003714 | 0.017771 | 0.209 | -0.83466 |
| progressive Disease Duration (centred) | 0.026790 | 0.028882 | 0.928 | 0.35469 |
| depression | -0.777588 | 0.271821 | -2.861 | 0.00465 ** |
|  |  |  |  |  |
| Interaction term | 0.007371 | 0.017977 | 0.410 | 0.68220 |

**Significance codes: *** p < 0.001, ** p < 0.01, * p < 0.05

#### Model Performance

- **Residual Standard Error**: 1.764 on 210 degrees of freedom
- **Multiple R-squared**: 0.1909
- **Adjusted R-squared**: 0.1678
- **F-statistic**: 8.26 on 6 and 210 DF
- **p-value**: 4.878e-08
- **AIC**: 871.0242
- **BIC**: 898.0633

#### Model Fit and Assumptions

- **Residuals**: The distribution of the residuals was examined through plots and histograms. The residuals appeared to be approximately normally distributed, with the following summary statistics:
  - **Minimum**: -4.3345
  - **1st Quartile**: -1.2545
  - **Median**: -0.2242
  - **3rd Quartile**: 1.0927
  - **Maximum**: 4.5239


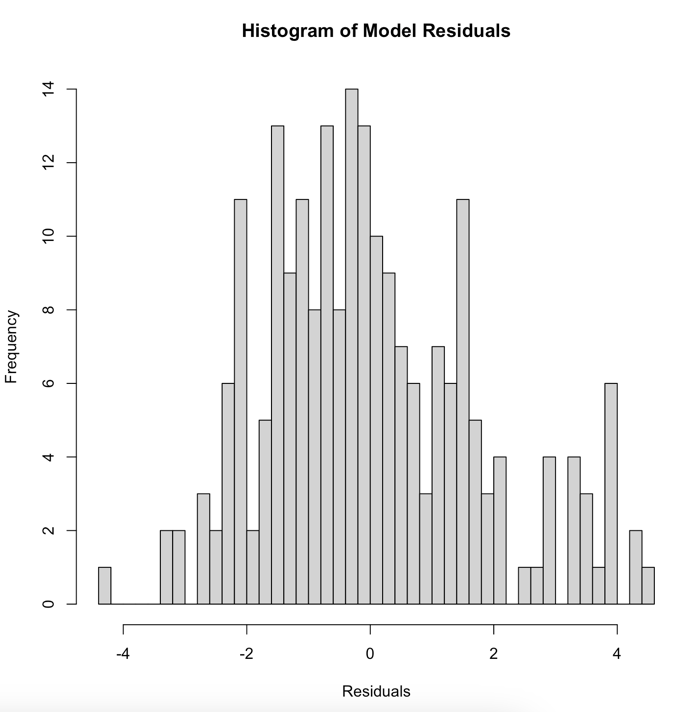

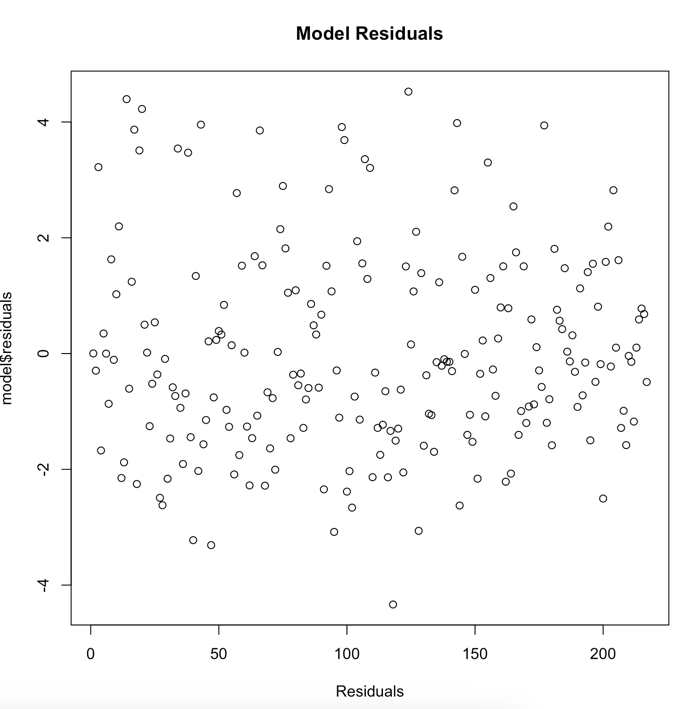


- **Generalised Variance Inflation Factor (VIF)**: Multicollinearity was assessed using VIF values, all of which were below 5, indicating no significant multicollinearity among the predictors:

|  | GVIF | Df | GVIF^(1/(2*Df)) | Interacts With |
| --- | --- | --- | --- | --- |
| Average Combined NHPT | 1.082502 | 3 | 1.0133 | EDSS ≥6 |
| EDSS ≥6 | 1.082502 | 3 | 1.0133 | Average Combined NHPT |
| Age (centred) | 1.106807 | 1 | 1.052049 | NA |
| Progressive disease duration (centred) | 1.103195 | 1 | 1.050331 | NA |
| Depression | 1.020092 | 1 | 1.009996 | NA |
