# Supplementary material for: The ABILHAND‐23 Patient Reported Outcome Measure in Secondary Progressive Multiple Sclerosis: A Cross‐Sectional Analysis With the Nine Hole Peg Test
Source: Brain Behav. 2025 Nov 26;15(12):e71101. doi: 10.1002/brb3.71101 (PMC12657254; doi:10.1002/brb3.71101)
Supplement: Supplementary file 2 — Supplementary Table: brb371101‐sup‐0002‐Table.docx [file BRB3-15-e71101-s001.docx]

| Study/Trial | Name | Intervention | PROM Version | ABILHAND Outcome | Clinical Outcome |
| --- | --- | --- | --- | --- | --- |
| Medicinal Trial | ASCEND (2018)  NCT01416181  DOI: 10.1016/ S1474-4422(18)30069-3  n=889 (SPMS)  Duration: 4 years | Natalizumab | ABILHAND-56  (3-responses) | Mean change 0 to 96w  Placebo: -3.45  (SD 14.739)  Treatment: -2.44  (SD 13.023)  (p=0.2586) | Confirmed composite-CDP  OR 0.86, 95% CI 0·66–1.13  (*p* = 0.286)  9HPT Progression  OR 0·56, 95% CI 0·40–0·80  (*p* = 0·001) |
|  | ENHANCE (2019)  NCT02219932  n=633,  (RR: 324, SP: 194, PP: 86, PR: 29)  Duration: 24 weeks | Fampridine | ABILHAND-56  (3-responses) | Mean change 0 to 24w  Placebo: +0.75 (SE: 0.593)  Treatment: +1.49 (SE: 0.574)  (p=0.197) | MSWS-12 ≥10 point improvement  OR 1.61, 95% CI 1.15–2.26  *p* = 0.006  TUG >15% improvement  OR 1.46; 95% CI 1.04–2.07  *p* = 0.03  No UL clinical outcome measures |
|  | INSPIRE  NCT02430532  (terminated) | DMF | ABILHAND-56  (unknown) | N/A  Terminated | N/A |
|  | CHARIOTMS (2027)  NCT04695080  n=200 (Any with EDSS 6.5 – 8.5)  Duration: 24 months | Cladribine | ABILHAND-56  (4-responses) | Ongoing  Targeted completion: 2027 | N/A |
| Rehabilitation | EMPOW (2024)  NCT05895734  DOI: 10.1186/s12984-024-01325-w  n=25 (RR: 10, SP: 12, PP: 3)  Duration: 8 weeks | Powerball^®^ and Conventional Physiotherapy | ABILHAND-23  (3-responses) | Mean difference after 8w of treatment:  0.25 (SD = 8.44)  *p* = >0.999 | 9HPT more affected limb mean difference in treatment vs controls after 8w:  -4.79 vs -2.5 (*p* = 0.077)  9HPT less affected limb mean difference in treatment vs controls after 8w:  -4.79 vs -2.5 (*p* = 0.077) |
|  | NCT04891341 (2023)  DOI: 10.1016/j.msard.2023.104558  n=34 (RR: 15; SP: 15; PP: 4)  Duration: 8 weeks | Telerehabilitation and Circuit Training | ABLIHAND-23 (3-responses) | Telerehabilitation benefit from pre-training 41.40 ± 5.73 to post-training 42.6 ± 4.75 (*p =* 0.04).  V-TOCT benefit from pre-training 39.29 ± 9.70 to post-training 43.53 ± 2.5 | Utilised the Minnesota Manual Dexterity Test (MMDT) and found significant benefit for both rehabilitation options. Did not utilise the 9HPT. |
|  | NCT04325074 (2022)  DOI: 10.5014/ajot.2022.044479  n=35  Duration: 3 months | Mental Practice +/- Skills Training | ABILHAND-23  (3-responses) | Reported benefit from all rehabilitation; details not available | Reported benefit from all rehabilitation; details not available |
|  | I-TRAVLE (2018)  NCT01918748  DOI:10.1080/17483107.2016.1278467  n=13 (RR: 5; SP: 6; PP: 2)  Duration: 3 months | Robot-mediated training | ABILHAND-23  (3-responses) | No significant before/after effect on ABILHAND (before 0.5 [-1.3 to 1.8] to after 0.9 [-0.6 to 1.8], nor on 3-month follow up (0.1 [-1.2 to 1.3]) | Sustained benefit in motor test of speed post-treatment and at 3-months, but no use of 9HPT |
|  | NCT04171908 (2020)  DOI: 10.1186/s12984-020-00718-x  n= 30 (RR: 11, SP: 13, PP: 6)  Duration: 10 weeks | sEMG controlled arm-band video games | ABILHAND-23  (3-responses) | Mean difference at 8 weeks:  3.67 (95% CI -3.79 to 11.3)  (p=0.322) | 9HPT more affected limb median difference in treatment vs controls at 10w:  -3.32 vs +9.93 (*p* = 0.069)  9HPT less affected limb median difference in treatment vs controls at 10w:  -1.55 vs -4.19 (*p* = 0.160) |
|  |  |  |  |  |  |
| Observational | MuSicalE (2025)  NCT03593590  n=1710 (RMS & PPMS)  Duration: 4 years | Ocrelizumab | ABILHAND-56  (unclear) | Ongoing  Targeted completion 2025 |  |
